# Circuit Techniques for High Efficiency Piezoelectric Energy Harvesting

**DOI:** 10.3390/mi13071044

**Published:** 2022-06-30

**Authors:** Yi Yang, Zhiyuan Chen, Qin Kuai, Junrui Liang, Jingjing Liu, Xiaoyang Zeng

**Affiliations:** 1 State Key Laboratory of ASIC and System, Fudan University, Shanghai 201203, China; 20112020087@fudan.edu.cn (Y.Y.); liujingjing@fudan.edu.cn (J.L.); 2 Novosense Microelectronics Company Ltd., Shanghai 200120, China; qin.kuai@novosns.com; 3 School of Informaiton Science and Technology, ShanghaiTech University, Shanghai 201210, China; liangjr@shanghaitech.edu.cn

**Keywords:** piezoelectric energy harvesting techniques, impedance matching, non-linear methods, AC-DC rectifiers, energy extraction and voltage flipping techniques, maximum power point tracking

## Abstract

This brief presents a tutorial on multifaceted techniques for high efficiency piezoelectric energy harvesting. For the purpose of helping design piezoelectric energy harvesting system according to different application scenarios, we summarize and discuss the recent design trends and challenges. We divide the design focus into the following three categories, namely, (1) AC-DC rectifiers, (2) C_P_ compensation circuits, (3) maximum power point tracking (MPPT) circuits. The features, problems encountered, and suitable systems of various AC-DC rectifier topologies are introduced and compared. The important role of non-linear methods for piezoelectric energy harvesting is illustrated from the perspective of impedance matching. Energy extraction techniques and voltage flipping techniques based on inductors, capacitors, and hybrid structures are analyzed. MPPT techniques with different features and targets are discussed.

## 1. Introduction

**Figure 1:**
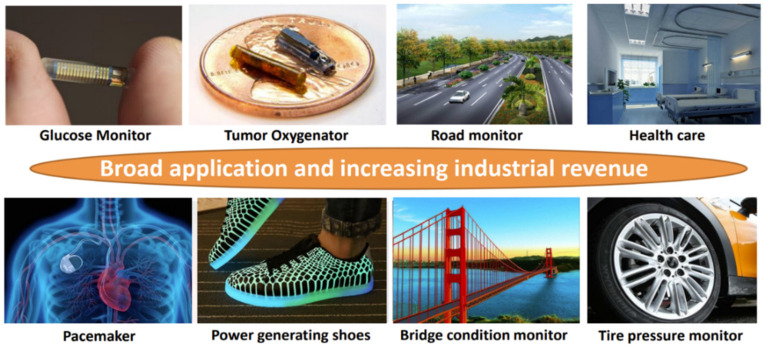
The target applications of micro energy harvesting.

In recent years, harvesting energy from solar [1], thermal [2], vibration [3] and etc. becomes an attractive solution, as it enables an autonomous and sustainable operation of numerous wireless devices. The use of piezoelectric energy harvesting technology to convert vibration energy into electrical energy has received extensive attention due to the advantages of high energy density and ease of integration. It can be applied to blood glucose monitor, tumor oxygenator, road monitoring, tire pressure monitoring and other scenarios [4,5,6,7,8,9], as shown in Figure 1.

**Figure 2:**
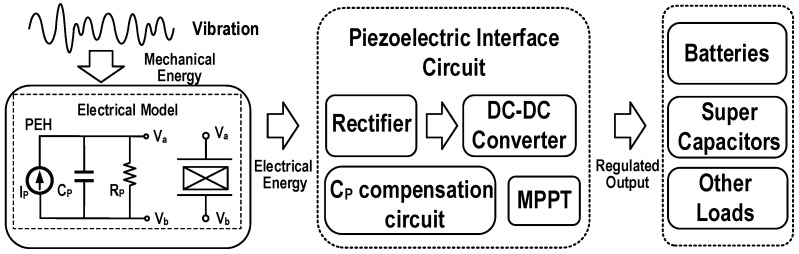
Block diagram of the piezoelectric energy harvesting system.

Piezoelectric energy harvester (PEH) generally adopts a mechanical cantilever structure composed of piezoelectric materials such as lead zirconate titanate [10]. The input vibration applied to the piezoelectric material will generate mechanical strains in the device, which can be converted to 10 μW–10 mW of available electrical power. A typical piezoelectric energy harvesting system is shown in Figure 2. The mechanical energy generated by vibration is converted into the electrical energy in the PEH, and then supplied to batteries, super capacitors or other loads through an interface circuit. A complete piezoelectric interface circuit consists of four parts: an AC-DC rectifier, a maximum power point tracking (MPPT) module, a dc-dc converter, and a *C_P_* compensation circuit.

The PEH generates an AC voltage when it vibrates, thus an AC-DC rectifier is required to convert AC voltage into DC voltage available for load. The power efficiency and voltage conversion efficiency of the rectifier are negatively affected by the path voltage drop, meanwhile the rectifier may encounter reverse current, leakage current, voltage oscillation and self-startup problems. Various rectifier topologies [11,12,13,14,15,16,17,18,19,20,21,22,23,24] were proposed and utilized to reduce the voltage drop and solve these problems, guaranteeing the correct rectification function and high efficiency. Due to the inherent capacitor *C_P_*, direct impedance matching to the PEH requires a large inductor, which is impractical in space-constrained applications. Utilizing the *C_P_* compensation circuit consists of synchronous switches and other components can keep the source voltage and current in the same direction, which is called non-linear methods [25,26]. Most of the non-linear methods can be grouped into two categories, the synchronous electric charge extraction (SECE) [17,18,19,27,28,29,30,31,32,33,34] and the synchronized switch harvesting (SSH) [1,10,14,15,16,22,25,35,36,37,38,39,40,41]. The research on them have mainly revolved around how to flip or extract the voltage across the PEH terminals, and some of them have realized the full integration for the deep issue implant systems [1,36]. To ensure the maximum output power, the general MPPT modules obtain the optimal output impedance by adjusting the parameters of the DC-DC converters [24,26,37,42,43,44,45]. In addition, the targets of some other MPPT circuits are specific according to the system characteristics. For example, in the multi excitation synchronous charge extraction (MCE) [17,28], MPPT is realized by ensuring that the energy extracted each time is the same.

In this brief, we systematically summarize the key aspects of impedance matching, AC-DC rectifiers, non-linear methods and MPPT circuits in the piezoelectric energy harvesting. In addition, we review the advantages and disadvantages of different methods or topologies, the challenges that can be solved, and the applicable piezoelectric energy harvesting systems.

## 2. Circuit Model and Impedance Matching

### 2.1. Piezoelectric Energy Harvester Circuit Model

**Figure 3:**
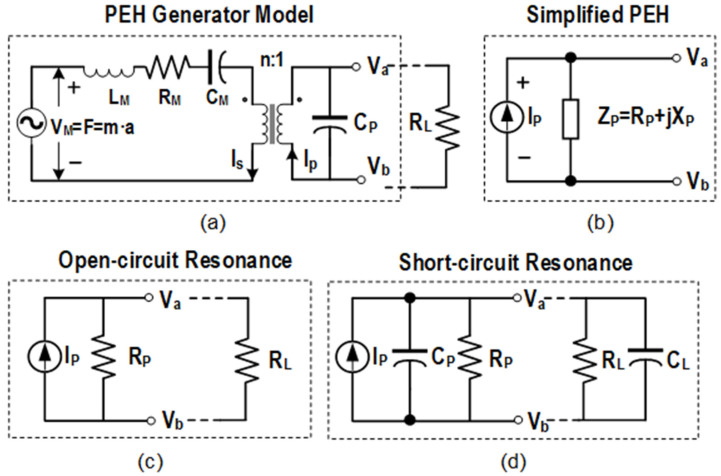
(**a**) Mechanical and electrical domain coupled models for piezoelectric energy harvesters, (**b**–**d**) Equivalent circuit of piezoelectric energy harvester when it vibrates at full frequency range, open-circuit resonance frequency and short-circuit resonance frequency, respectively.

**Figure 4:**
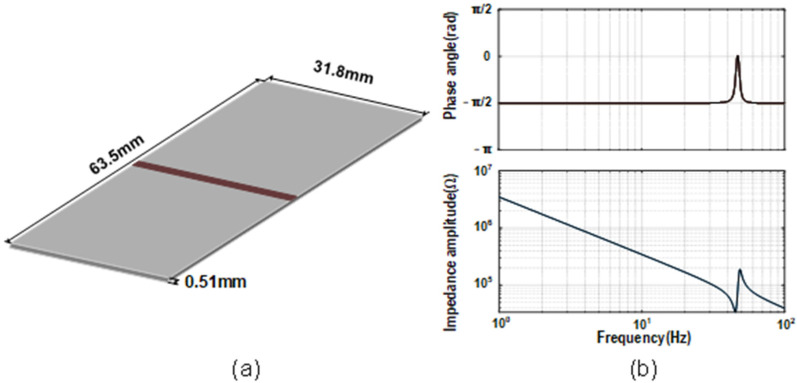
(**a**) Dimensions, (**b**) source impedance of the piezoelectric harvester T226-A4-503X.

When the PEH is subjected to mechanical vibrations, the piezoelectric material is stressed to generate an induced electromotive force. It can be regarded as a mechanical spring mass system coupled to an electrical domain and a mechanical domain [46], as shown in Figure 3a. In the mechanical domain, *V_M_* represents the excitation at a certain vibration acceleration, *L_M_* represents the mechanical mass, *R_M_* represents the mechanical losses, and *C_M_* represents the reciprocal of mechanical stiffness. The electromechanical coupling is modeled as a transformer with turn ratio n. In the electrical domain, Figure 3b shows that the PEH can be equivalent to the parallel connection of a current source *I_P_* and an impedance *Z_P_*. *Z_P_* is the total source impedance obtained by combining *C_P_* with *L_M_*, *C_M_*, and *R_M_* converted by the transformer. Figure 4 depicts the dimensions and source impedance of the bimorph piezoelectric harvester T226-A4-503X with parameters in Table 1, as a representative piezoelectric element for demonstration [26].

### 2.2. Impedance Matching

To maximize energy extracted from the PEH, impedance matching between the load and source should be achieved. The source impedance of the PEH can be expressed as,

(1) $$Z_{P}=R_{P}+jX_{P}.$$

When the load impedance *Z_L_* is equal to the conjugate of the source impedance, expressed as *Z_L_* = *R_P_* − *jX_P_*, the maximum output power can be extracted. This method is called conjugate matching [47]. The output power to achieve conjugate matching can be obtained as,

(2) $$P_{CON}={\left|\frac{Z_{P}}{Z_{P}+Z_{L}}\right|}^{2}I_{P}{}^{2}Z_{L}=\frac{R_{P}{}^{2}+X_{P}{}^{2}}{4R_{P}}I_{P}{}^{2}.$$

However, it is impractical to implement the conjugate matching directly because a very large inductor is required. Matching the load resistance with source impedance is called resistance matching [26], which needs the load resistance *R_L_* equal to $\sqrt{R_{P}{}^{2}+X_{P}{}^{2}}$. The maximum output power of resistive matching can be obtained as,

(3) $$P_{RES}=\frac{R_{P}{}^{2}+X_{P}{}^{2}}{2\left(R_{P}+\sqrt{R_{P}{}^{2}+X_{P}{}^{2}}\right)}I_{P}{}^{2}.$$

Figure 3c depicts the circuit model of the PEH when it vibrates at the open-circuit resonance frequency. In this situation, the source impedance *Z_P_* becomes purely resistive [26]. When the load resistance *R_L_* is equal to the source resistance *R_P_*, the maximum output power can be obtained as,

(4) $$P_{RES}=\frac{I_{P}{}^{2}}{4R_{P}}\;.$$

Note that resistance matching has very strict frequency requirements. When the frequency shift occurs, as the ratio of the imaginary part to the real part of the source impedance increases, the efficiency of resistance matching becomes worse than that of conjugate matching.

**Figure 5:**
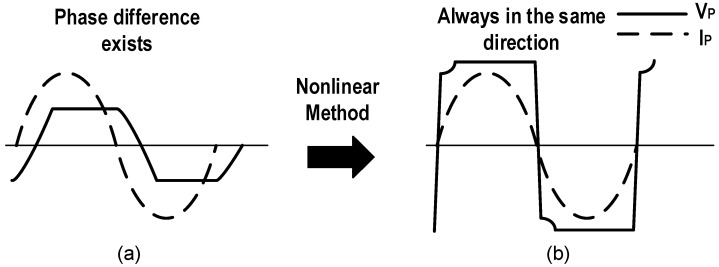
(**a**) Original waveforms of *V_P_* and *I_P_*, (**b**) waveforms of *V_P_* and *I_P_* after non-linear processing.

When the PEH vibrates at the short-circuit resonance frequency, as shown in Figure 3d, it can be modeled as the parallel connection of a current source *I_P_*, a resistor *R_P_* and the inherent capacitor *C_P_*. The source impedance can be approximated as purely capacitive since *R_P_* ≫ 1/(*ωC_P_*), meanwhile it is capacitive except for the open-circuit resonance frequency. In this situation, the open circuit voltage of the model is *I_P_*/(*ωC_P_*), which is much smaller than the product of *I_P_* and *R_P_*, so the corresponding output power is greatly reduced. That is caused by the phase difference between *V_P_* and *I_P_*, as depicted in Figure 5a. The accumulated charge in *C_P_* needs to be neutralized, resulting in a large amount of energy loss. To solve the problem, researchers utilized non-linear methods to shape the voltage and current waveforms by controlling synchronous switches, which makes them always in the same direction as shown in Figure 5b. As a result, high output power can be obtained in a wide frequency range.

## 3. Rectifiers

### 3.1. Rectifier Models

**Figure 6:**
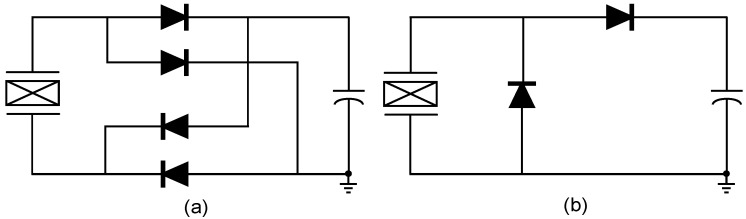
Structure diagrams of (**a**) full bridge rectifier and (**b**) voltage doubler.

The interface circuit needs to convert AC voltage generated by the PEH to the DC voltage by rectifiers while delivering as much energy as possible to the load. Full-bridge rectifier (FBR) [48,49] and voltage doubler [50,51] are commonly used rectifiers due to their simplicity and ease of integration. Figure 6 depicts their structure diagrams. The direct use of the FBR and doubler as an interface circuit has large energy loss due to the phase difference between *V_P_* and *I_P_*. Therefore, the FBR usually plays a rectifying role in the high efficiency piezoelectric energy harvesting interface circuits using non-linear methods. The voltage doubler can also be utilized for the detection of the open-circuit voltage [37]. Since the performance of the FBR determines whether the piezoelectric energy harvesting system can work properly, we will focus on its implementation and related improved circuits in the next section.

### 3.2. Active Implementations of FBR

**Figure 7:**
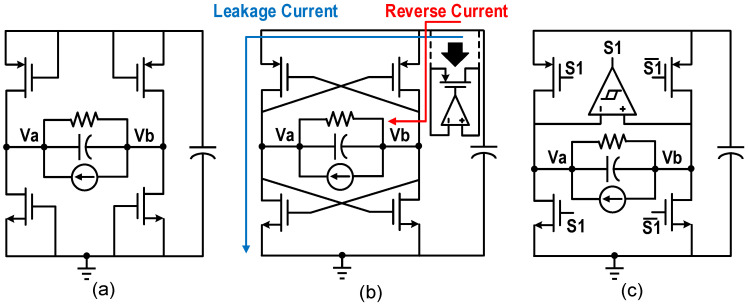
Structure diagrams of (**a**) diode-connected rectifier, (**b**) negative voltage converter, (**c**) extraction efficiency enhanced rectifier for the SECE.

The simplest active implementation of the FBR consists of four diode-connected MOSFETs, as shown in Figure 7a. However, the existence of the forward voltage drop greatly reduces the output power. To improve the efficiency of the rectifier, Xu et al. proposed a negative voltage converter (NVC) [11]. As shown in Figure 7b, the structure consists of a pair of cross-coupled NMOS and a pair of cross-coupled PMOS. However, two problems need to be solved for NVC. First, in the process of re-establishing the PEH voltage after the current direction changes, two NMOS are simultaneously turned on for a long time, so that *V_RECT_* is limited to the NMOS threshold voltage. Moreover, a leakage current from *V_RECT_* to the ground will be formed. NVC can be protected from these by rapidly flipping the voltage of the PEH through an inductor or capacitor array. Second, when the voltage at the higher terminal of the PEH is less than *V_RECT_*, a reverse current is formed from the output to the PEH, causing a large amount of energy loss. Adding an active diode between NVC and the output capacitor can effectively reduce the reverse current [14,15,16], but an additional switch will increase the on-resistance of the path, which has a negative impact on the output efficiency.

Since the SECE operates in an open-circuit state most of the time, its output efficiency does not suffer from above-mentioned problems [27]. However, during the process of energy transfer, the on-resistance of the NMOS increases with declining the gate voltage, increasing the conduction losses of *LC* resonance. More seriously, the path is disconnected when the voltage across the PEH drops to the threshold voltage, resulting in an incomplete extraction of energy. It has a negative effect on the output power, especially under weak excitation. It can be effectively solved by controlling the switches by a hysteretic comparator with constant output voltage during the energy extraction process [19], as shown in Figure 7c.

**Figure 8:**
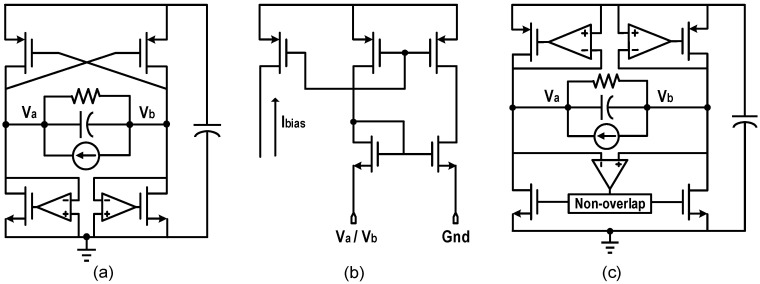
Structure diagrams of (**a**) comparator-controlled active rectifier (**b**) op-amp for active rectifier (**c**) fully comparator-controlled rectifier.

Figure 8a depicts the structure diagram of the comparator-controlled active rectifier [12]. Compared to NVC, unbalanced comparators are used instead to control NMOS, forming active diodes. The reverse current is greatly suppressed without an additional switch in the path. However, it is difficult for the comparator to turn off the NMOS accurately when the current crosses zero. This will lead to the following two problems. First, before the direction of *I_P_* changes, the comparator will turn off the NMOS prematurely. Then the current will continue to charge *C_P_*, making the voltage at the negative terminal of the comparator begin to drop and therefore the path is opened again. The cycle of this process causes the voltage to oscillate. Second, when the higher end voltage of the PEH is less than *V_RECT_*, if the NMOS is not turned off in time, it will lead to the reverse current and output power loss. Adding an offset voltage to the input terminal of the comparator is a common solution to these problems [20]. However, process variations will cause errors in the offset voltage. Due to its high magnification, replacing the comparator with an operational amplifier shown in Figure 8b can more accurately turn off the NMOS by monitoring its *V_DS_* [13]. Chang et al. proposed the fully comparator-controlled rectifier to solve the problems of the oscillation and reverse current [21]. Figure 8c shows the new topology, the cross-coupled PMOS were replaced with comparator-controlled active diodes, and a comparator with the non-overlapping circuit were responsible for con trolling the NMOS.

### 3.3. Expansions of Active Rectifiers

**Figure 9:**
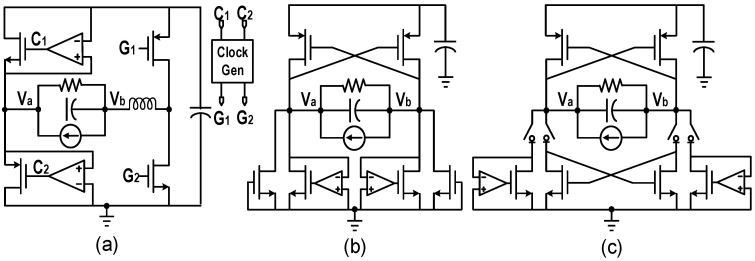
Structure diagrams of (**a**) active rectifier for the automatic control of voltage flipping (**b**) start-up optimized active rectifier (**c**) dual-mode active rectifier.

Wu et al. utilized signals G_1_ and G_2_, generated by comparator output signals C_1_ and C_2_ through a clock generator to control switches other than active diodes [22]. Figure 9a depicts the structure diagram. It not only realized the function of the active rectifier, but also achieved the automatic control of voltage flipping based on inductor without adjusting the switching time.

Although the comparator/operational amplifier controlled active rectifier can effectively reduce the impact of leakage current and reverse current on the output efficiency, it may not work properly during the startup process in self-powered systems. To solve the problem, Lu et al. added diode-connected NMOS in parallel to each active diode [23], as shown in Figure 9b. The diode-connected NMOS worked instead of the active diodes during startup, and were shorted by the active diodes during the normal operation of the rectifier. However, the improved rectifier suffered from slow startup speed and low output efficiency especially with weak excitation due to the large voltage drop of the diode-connected NMOS. To improve the startup speed, a dual-mode rectifier was proposed in [24]. As shown in Figure 9c, the diode-connected NMOS were replaced by cross-coupled NMOS, and four switches were added to control the mode of the rectifier. Since the on-resistance of the NMOS decreases with the increase of the gate voltage during startup process, measurement results showed that the cross-coupled NMOS with 25 μm width had better conversion efficiency than the diode-connected NMOS with 800 μm width [24]. However, the power efficiency in the normal mode will be slightly reduced due to the extra switches in the path.

### 3.4. Performance Comparison between Different Rectifier Topologies

Table 2 compares the voltage drop, power efficiency and voltage conversion ratio (*V_RECT_*/*V_P_*) of different rectifiers. The voltage drop on the main path of the rectifier greatly affects the efficiency of the rectifier, so it is the main comparison index. Diode connected FBR ([10]) has high stability and robustness compared with other topologies. By using cross coupled transistors, the voltage drop of [11] is reduced from 2 *V_TH_* to 3 *V_DS_*. Although an additional active diode is added, the voltage conversion ratio and power efficiency of are significantly improved. Since no additional active diode is required, the voltage drop of the active diode controlled by the comparator becomes 2 *V_DS_* [12]. Compared with the comparator circuit, the operational amplifier control circuit [13] has a higher amplification factor, which can more accurately close the path when the reverse current is generated. Adding an additional comparator and non-overlapping circuit to control a couple of MOSFETs can further reduce the reverse current and increase the power efficiency, but extra static power is added [21]. By adding the cross-coupled NMOS, self-start and high energy efficiency can be realized simultaneously [24].

## 4. Non-Linear Methods

### 4.1. Synchronous Electric Charge Extraction

**Figure 10:**
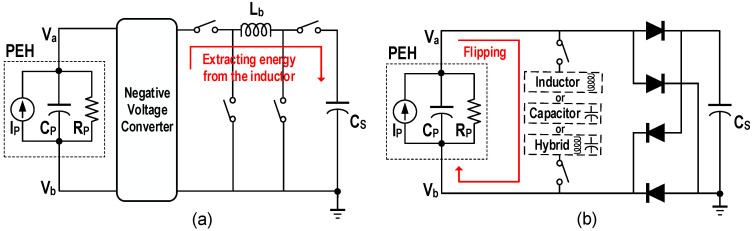
Interface circuit architecture diagrams of (**a**) the SECE, (**b**) the SSH.

The operating principle of the SECE [27] is that when *I_P_* crosses zero, the switches are turned on and the inductor transfers the energy accumulated in *C_P_* to the storage capacitor *C_S_*, as shown in Figure 10a. Compared with the SSH, the SECE has the advantage that the output power is independent of the load voltage as it most often exists in an open-circuit state. However, a large amount of energy will be consumed by the series resistance of the inductor in the process of energy transfer especially when the inductance value is small, which is called conduction losses. In [27], when the PEH operated at resonance, the simulated conduction losses occupied 90.1% of the total power consumption, and the maximum power efficiency of the system in the measurement was 85%. To reduce the conduction losses, the MCE was proposed in [28]. By utilizing a multiple extraction strategy, the peak current of the inductor during energy transmission was divided by $\sqrt{N}$. The experimental results showed that the conduction losses were also approximately divided by $\sqrt{N}$. Pre-charge is another technique to increase the output power of the SECE [29]. In contrast with the multiple extraction technique, which increase the transmission efficiency by reducing energy loss, it increases the amount of energy that can be extracted (*E_EXT_*). The specific operation is to pre-charge the voltage of *C_P_* to *V*_1_ after the extraction process. It can be realized by utilizing the inductor to flip the residual voltage of *C_P_* or transfer a part of energy back from *C_S_*. Assuming under ideal conditions, *V_P_* can reach *V*_1_ + *V_P_*_0_ at the next zero-crossing of *I_P_*, *V_P_*_0_ is the maximum value for the voltage of *C_P_* without pre-charge. Furthermore, the increase in *E_EXT_* can be obtained as,

(5) $$\Delta E_{EXT}=0.5C_{P}\left({\left(V_{1}+V_{P0}\right)}^{2}-{\left(V_{1}\right)}^{2}-{\left(V_{P0}\right)}^{2}\right)=C_{P}V_{P0}V_{1}.$$

**Figure 11:**
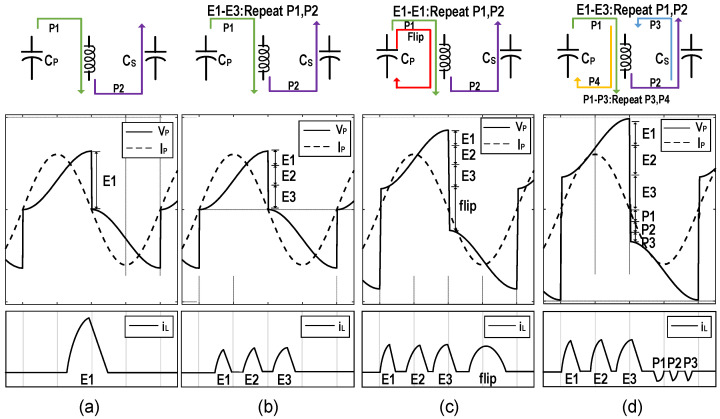
Schematic diagrams of energy transferred path and waveforms of *V_P_*, *I_P_*, and *i_L_* for (**a**) the SECE, (**b**) the MCE, (**c**) the MCEBF and (**d**) the MCE-MPC interface.

On the basis of the multiple extraction and the pre-charge techniques, the multiple charge extraction with bias-flip (MCEBF) was proposed [30], which flipped the residual voltage after completing multiple extraction. Apparently, the conduction losses during pre-charge process can also be reduced by transfer the energy multiple times. Moreover, the extent of pre-charge has the effect on the increase in the output power. Therefore, the multiple charge extractions and multiple pre-charge (MCE-MPC) was proposed [31] to further improve the output capacity of the system. It utilized the energy of *C_S_* for pre-charge, which enabled the multiple transfer and precise control of the pre-charge energy. The simulation results shown the output power of the MCE-MPC was increased by 101.92%, 24.60%, and 7.62% compared with the SECE, MCE, and MCE-MPC, respectively. Figure 11 depicts their energy transfer paths, the waveforms of *V_P_*, *I_P_* and the inductor current *i_L_* for demonstration.

### 4.2. Synchronized Switch Harvesting

**Figure 12:**
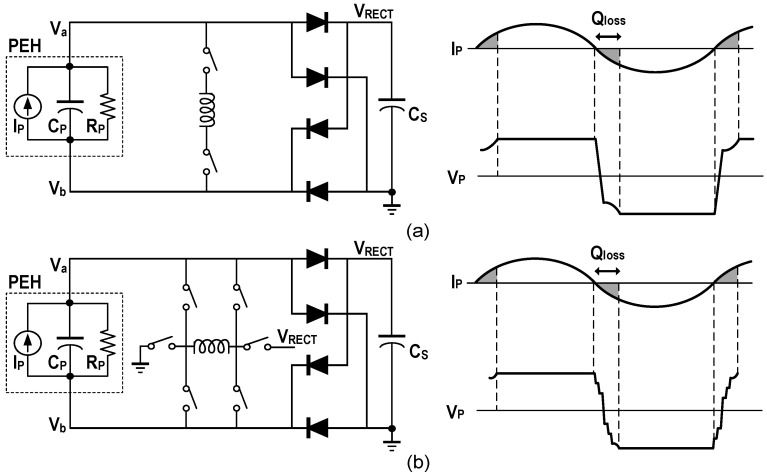
Interface circuit structures and *I_P_*, *V_P_* waveforms diagrams of (**a**) the SSHI, (**b**) the multi-step flipping SSHI, respectively.

Compared with the SECE, the SSH does not change the output mode of the FBR and usually has higher output power. As shown in Figure 10a, it can be based on an inductor, capacitors and a hybrid structure. The synchronous switch harvesting on inductor (SSHI) is the most popular structure among them. Its interface circuit and related waveforms are shown in Figure 12a. When the current direction changes, the inductor is utilized to flip the voltage of *C_P_*, which greatly shortens the time to establish an effective output. The SSHI has the advantages of high output power, low complexity and high maximum output power improving rate (MOPIR) compared to the FBR. In [14], the output power of 408 μW and 4.83× MOPIR at resonance was obtained with a 3.3 mH inductor. As shown in Figure 12b, a multiple flipping strategy can also be utilized with the help of *C_S_*, and 4.48× MOPIR was realized with a 47 μH inductor [16]. However, there are also some challenges for the SSHI. The switching time for *LC* resonance needs to be precisely controlled, especially for the multiple flipping. More seriously, the inductor occupies a large volume, so it is not suitable for the application scenarios with space-constraints.

The piezoelectric energy acquisition system of deep tissue is powered by external ultrasonic source. Due to specific application requirements, the size of implants is usually limited to ~10 mm^3^, and the limited power is less than 100 μW. Minimizing or even no external components are the most important limitation. To satisfy this requirement, capacitors were utilized to flip the voltage of *C_P_* [1,35,36,37]. Some of the related work was based on on-chip capacitors, enabling the full integration of the system [1,36].

**Figure 13:**
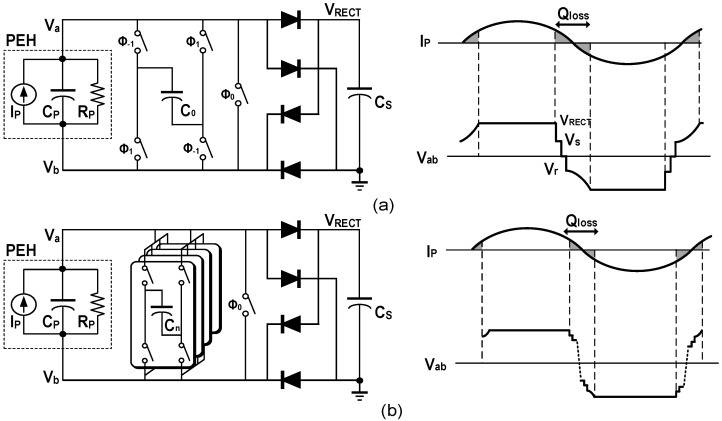
Interface circuit structures and *I_P_*, *V_P_* waveforms diagrams of (**a**) flipping with single capacitor, (**b**) flipping with capacitor array, respectively.

Figure 13a shows the basic form based on a single capacitor with five switches and illustrates the corresponding voltage and current. When *I_P_* crosses zero, the charge of *C_P_* is redistributed to *C*_0_ and then *C*_0_ recharges *C_P_* in the opposite direction. After finishing these two processes, the voltage of *C_P_* is defined as vs. and *V_r_*, respectively. According to the charge conservation law, the charge balancing equations can be expressed as,

(6) $$(C_{P}+C_{0})V_{s}=C_{P}V_{rect}+C_{0}V_{r},$$

(7) $$C_{0}V_{s}=(C_{P}+C_{0})V_{r}.$$

**Figure 14:**
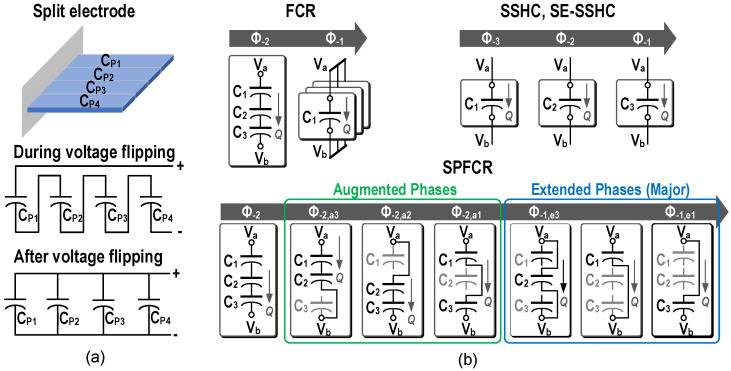
(**a**) Schematic diagram of the split electrode PEH, electrode region reconfiguration during and after the voltage flipping process, (**b**) capacitor reconfiguration examples using three flying capacitors for the FCR, the SSHC, the SE-SSHC, and the SPFCR.

From (6) and (7), the flipping efficiency $\eta_{flip}$ based on single capacitor can be derived as,

(8) $$\eta_{flip}=\frac{V_{r}}{V_{RECT}}=\frac{C_{0}}{C_{P}+2C_{0}}=\frac{1}{3},$$

when *C*_0_ equals to *C_P_*. $\eta_{flip}$ can be improved by utilizing capacitor array to flip, as shown in Figure 13b. In the synchronized switch harvesting on capacitors (SSHC) [35] proposed by Du et al., several parallel capacitors were utilized to share the charge with *C_P_* in turn, and then recharge *C_P_* in the opposite direction in reverse order. With eight off-chip capacitors all equal to *C_P_*, $\eta_{flip}$ increased to 80% and 9.3× performance improvement compared with the conventional FBR was obtained. Compared with SSHC, a PEH with its electrode split into several regions was utilized in split electrode SSHC (SE-SSHC) [36]. As shown in Figure 14a, by connecting these regions in series during the voltage flipping process, the effective capacitance of the PEH was reduced. As a result, the flipping efficiency of 69% was obtained with eight on-chip capacitors, and full integration of the system was realized. Both with on-chip capacitors, the voltage of *C_P_* was flipped by changing the topology of the capacitor array in the flipping-capacitor rectifier (FCR) [1]. Compared with sharing charge between *C_P_* and parallel flipping capacitors in order, higher flipping efficiency can be obtained with the same number of capacitors, although the control complexity was increased. To avoid the redistribution losses, the total capacitance of each topology should be equal. To further realize the compromise between the flipping efficiency and the number of capacitors, Chen et al. proposed the split-phase flipping-capacitor rectifier (SPFCR) [37]. It expanded the number of phases of the capacitor array in the FCR, and gave up the phases with lower efficiency. 9.3× MOPIR with *V_D_* = 0.12 V was realized compared with the conventional FBR for the SPFCR interface. Figure 14b shows the capacitor reconfiguration examples for the FCR, the SSHC, the SE-SSHC, and the SPFCR.

**Figure 15:**
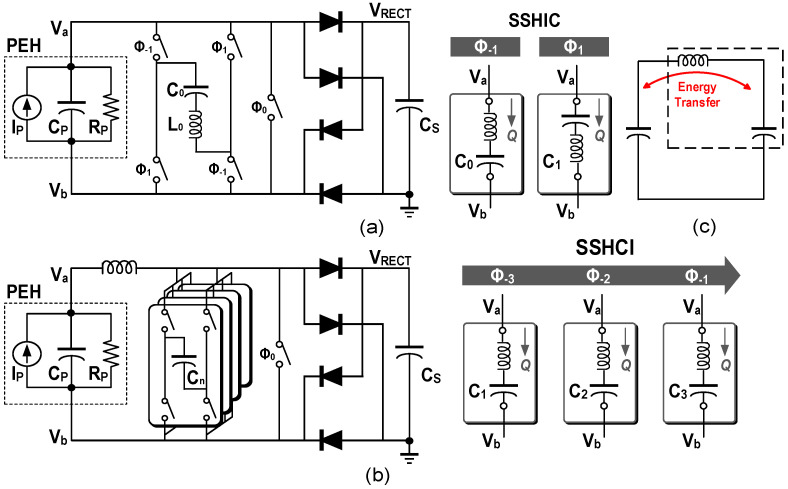
Interface circuit structures and hybrid reconfiguration examples of (**a**) the SSHCI, (**b**) the SSHIC. (**c**) Energy transferred model for hybrid flipping.

On the basis of previous work, researchers tried to combine the inductor and capacitor to flip the voltage which has potential for further improvement in the flipping efficiency and output power. Figure 15c shows the basic model of its energy transfer. The synchronized switch harvesting on capacitor-inductor (SSHCI) was proposed in [38]. As shown in Figure 15a, a capacitor *C*_0_ equal to *C_P_* was added in the *LC* resonance path and the flipping process of SSHI was divided into two phases. Through the inductor, the charge of *C_P_* was transferred to *C*_0_, and then transferred back to *C_P_* in the opposite direction at the second phase. 90.1% flipping efficiency was achieved with the inductance of 68 μH. The synchronized switch harvesting on inductor and capacitor (SSHIC) can be regarded as an extension of the SSHC [39], its interface circuit and phase reconfiguration are shown in Figure 15b. An inductor was added between *C_P_* and the parallel flipping capacitors to reduce the distribution losses. The simulation results showed 95.1% flipping efficiency and 1.22× power extraction compared with the SSHC can be achieved with 6 capacitors and a 2.2 mH inductor. Table 3 summarize the power levels, efficiencies, the pros and cons of some representative non-linear systems.

## 5. Maximum Power Point Tracking

The output power of the piezoelectric energy harvesting systems is affected by factors such as output impedance, phase shift, etc. Therefore, it is necessary to adjust these parameters according to the input excitation to ensure that the maximum output power can be obtained. This strategy is called maximum power point tracking.

### 5.1. General Types of MPPT

The commonly used MPPT methods can be roughly divided into two categories, perturb and observe (P&O) and fractional open circuit voltage (FOCV). The P&O is independent from the PEH characteristics and can also be applied to the MPPT for various targets. It receives the output power information by monitoring the voltage or current in the systems. By performing search algorithms such as climbing algorithm, parameters that affect the output power are adjusted until the circuit configuration with the maximum output power is obtained. In [26], a microcontroller unit (MCU) was utilized to perform a hill-climbing algorithm. Resistance matching was achieved by adjusting the switching frequency of a discontinuous conduction mode flyback converter. However, the MCU consumes a lot of time and power. In [42], a fully analog computer with a variable step size P&O was utilized to adjust a high voltage buck converter, which consumes much lower power and time than the MCU. In addition, with a fully analog computer, the SSHI with high tracking-efficiency MPPT were first demonstrated simultaneously in [43]. For interface circuits such as the FBR and the SSH, the system can operate in maximum power output state by setting *V_RECT_* to the maximum power point (*V_MPP_*), and *V_MPP_* can be directly obtained by detecting the open-circuit voltage. When the diode forward voltage is zero, *V_MPP_* is one-half the open circuit voltage. *V_OC_*_,*FBR*_ is the open circuit voltage of the FBR which equals to the original open-circuit voltage of the PEH. *V_OC_*_,*SSH*_, the open circuit voltage of the SSH, is obtained by flipping *V_OC_*_,*FBR*_ by the inductor or capacitor and increases with the flipping efficiency. The FOCV is a common method to achieve the maximum output power for the FBR, the SSH and other interface circuits utilizing the above rules. It generally includes two steps. First, disconnect the PEH and interface circuit to detect the open-circuit voltage. Second, adjust *V_RECT_* according to the input excitation to ensure the maximum output power. Compared with P&O, the FOCV has the advantages of low complexity, low power consumption and short setup time.

**Figure 16:**
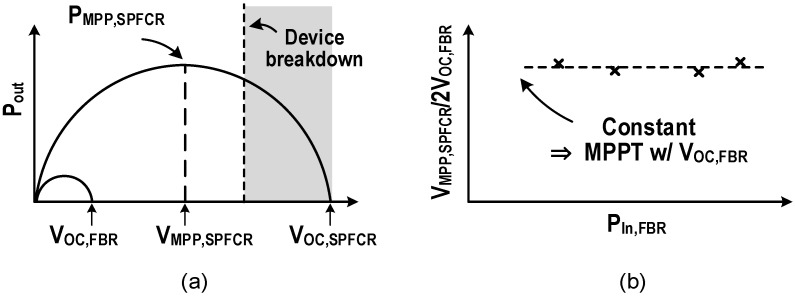
Illustrative diagram showing (**a**) relationship among *V_OC_*_,*FBR*_, *V_MPP_*_,*SPFCR*_, and *V_OC_*_,*SPFCR*_, (**b**) empirical constant ratio between *V_MPP_*_,*SPFCR*_ and *V_OC_*_,*FBR*_ over the FBR equivalent input power (Reprinted/adapted with permission from Ref. [37]. 2022, Zhiyuan Chen).

**Figure 17:**
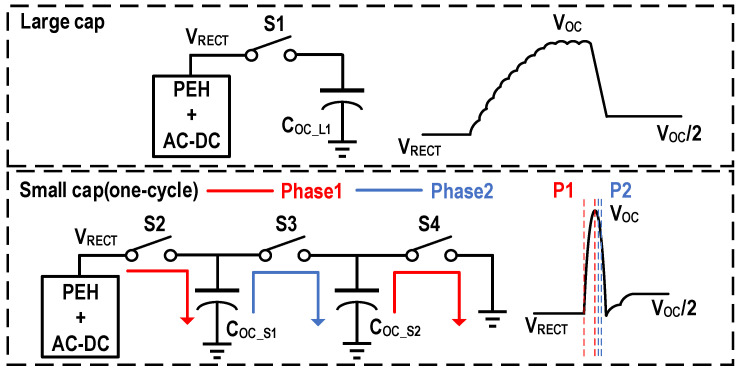
Comparison of the conventional FOCV and the one-cycle open-circuit voltage detection.

However, the open-circuit voltage of the high flipping efficiency interface circuits such as the SSHI, the SSHC, and the FCR may exceed the withstand voltage of CMOS. Therefore, it is difficult to be directly measured by commonly voltage detection circuits. Meanwhile, utilizing HV devices will increase the cost and reduce the stability of the system. In [37], it was verified by experiments that there is a fixed proportional relationship between the SPFCR maximum power point (*V_MPP_*_,*SPFCR*_) and *V_OC_*_,*FBR*_. The ratio is 1/(1 − $\eta_{flip}$), and the illustrative diagram is show in Figure 16. Therefore, *V_MPP_*_,*SPFCR*_ can be indirectly obtained to determine maximum power point by measuring *V_OC_*_,*FBR*_. The FOCV disconnects the PEH from the interface circuit when detecting the open-circuit voltage, so there is an output power loss during this time. An adaptive MPPT for the FBR was proposed [44], which was also based on the characteristic that the FBR operates at the maximum power point when *V_RECT_* equals to 1/*2V_OC_*_,*FBR*_. It connected a high pass filter at the output of the rectifier. When *V_RECT_* increased to 1/*2V_OC_*_,*FBR*_, the resistor voltage of high pass filter reached the maximum. The DC-DC convertor for output was thus turned on, and V_RECT_ can be kept at 1/2*V_OC_*_,*FBR*_ adaptively when excitation changed without the disconnection for detection. Similarly, according to the law that there is a certain proportional relationship between *V_RECT_* and the maximum value of the PEH voltage, an adaptive MPPT based on the series-SSHI was proposed in [40]. However, the adaptive MPPT has not been fully proven to be applicable to other interface circuits. Therefore, shortening the detection time may be a more adaptive solution for reducing the power loss. A large sensing capacitor is utilized to reduce the voltage ripple in the conventional FOCV. *V_OC_* can be easily sensed but it takes several cycles. Through two small sensing capacitors and a peak detector, a one-cycle open circuit voltage detection was realized in [24]. When the voltage of the first sensing capacitor reached *V_OC_*, the peak detector briefly turned off the switch and obtains 1/2*V_OC_* value through charge sharing. After sensing, the 9.09 ms/V MPPT time was realized with a buck-boost converter controlled by a voltage multiplexer and low power ramp generator. Figure 17 depicts the comparison of the conventional FOCV and the one-cycle detection. In Table 4, performance, features, pros and cons of some P&O and FOCV work are listed.

### 5.2. MPPT for MCE

The MCE utilizes the strategy of multiple extraction to reduce the conduction losses in the energy transmission process of the SECE. It was proved that the energy of each extraction process should be equal to minimize the conduction losses [28]. The *N*th extraction time $T_{n}$ is obtained as,

(9) $$T_{n}=\frac{1}{\omega }arccos\left(\sqrt{\frac{N-n}{N-n+1}}\right)=\frac{a_{n}}{\omega },$$

$\omega =1/\sqrt{LC}$ and *N* is the number of the extraction process. In [28], each coefficient $a_{n}$ was stored in an 8-bit digital circuit, and the time of each extraction was set by controlling the number of the clocks to realize maximum power extraction. In [17], two energy sensing circuits and a comparator were utilized to realize the equal extraction of the energy. The first energy sensing circuit indicated the energy that needed to be extracted in a single time through a flipped voltage follower, current mirrors and *N* parallel capacitors. Then another energy sensing circuit with the similar structure detected the energy extracted each time. When the appropriate energy was extracted, the comparator ended the extraction and started the next extraction.

### 5.3. MPPT for Wide Frequency Range

**Figure 18:**
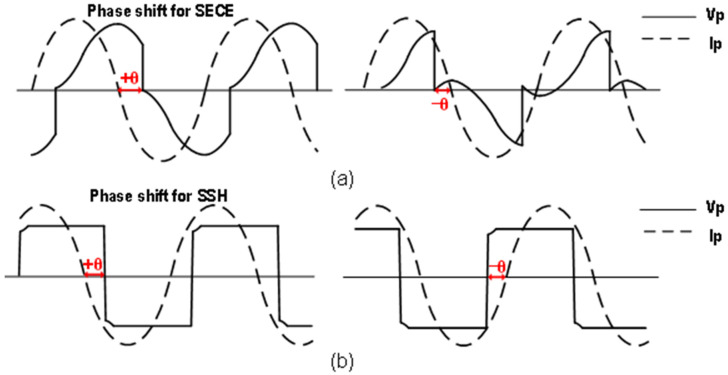
Illustrative diagram showing phase shift for (**a**) the SECE, and (**b**) the SSHI.

When the PEH vibrates off short-circuit resonance frequency, the series connection of *R_M_*, *L_M_* and *C_M_* no longer behaves purely resistive. Even if *V_P_* and *I_P_* are made in the same direction by the non-linear methods, the output power will drop due to the phase difference between *V_M_* and *V_P_*. Through theoretical analysis and experiments, it was demonstrated that adding phase shift between *V_P_* and *I_P_* had a positive effect on the output power of the SSHI and SECE operating off resonance frequency [32,41], as shown in Figure 18. Although a wide frequency operating range was achieved, these works were based on discrete devices or simulations. Meanwhile, the phase shift needed to be manually adjusted, so they were difficult to be utilized in practical applications. An automatic setting of the phase shift for the SECE was achieved in [33]. Compared to manual tuning, there was only an average power loss of 8.7% within the same bandwidth, while the system was self-powered. According to the variation of the optimal delay time t_1_ and energy extraction time t_2_ with frequency, the system operating range was divided into 5 frequency regions. After the frequency detector determined in which frequency region the PEH vibrated, the corresponding t_1_/t_2_ combination will be selected. However, the parameters of the PEH, frequency regions and t_1_/t_2_ combination needed to be known and predefined. The delay time of the SECE was adjusted by a 7b SAR-ADC in [34], and the optimal delay time was searched by the P&O algorithm. Compared with [33], the optimal delay time can be converged without relying on the PEH parameters and the 3-dB bandwidth was increased by three times.

### 5.4. MPPT for Multi-Input Single-Inductor Multi-Output (MISIMO) Systems

**Figure 19:**
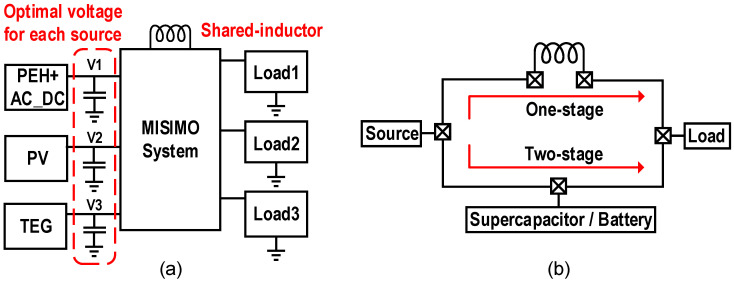
(**a**) System architecture of the MISIMO, (**b**) one/two-stage transfer schematic diagram.

Since the excitation for the PEH is determined by the vibration intensity, an upper limit exists for more efficient piezoelectric energy harvesting methods to increase the output power. Recently, the MISIMO energy harvesting system has been a research hotspot. Figure 19a shows the MISIMO system architecture, it harvests energy from different sources such as thermal, solar and vibration and supplies power to multiple loads. Compared to utilizing a PEH as a single input, the output power is further improved.

The maximum power point tracking for the MISIMO can be divided into two aspects. The first is to set the appropriate output impedance for each source based on its characteristics and excitation. The input sources in [52] were a PEH, a photovoltaic cell (PV) and a thermoelectric generator (TEG). The PEH and TEG were suitable for the conventional impedance matching model, and their MPPT were achieved by the FOCV. The MPPT of the PV was obtained by utilizing the hill-climbing algorithm with the current of a boost converter as the parameter to monitor. In [53], the MPPT of all three input sources including the PV were realized by the FOCV. Different from [53], a multi-step voltage-regulating SECE (MSVR-SECE) was utilized for the PEH in [54]. The operation principle of the MSVR-SECE was to perform the MCE when the excitation was above a set threshold, otherwise performed the SECE.

The energy transmission paths in the MISIMO system are divided into source-to-load, source-to-battery, and battery-to-source. The source-to-load is a one-stage transfer, and the set of the source-to-battery and battery-to-source is a two-stage transfer, the schematic diagram is shown in Figure 19b. Increasing the proportion of one-stage transfer can reduce the total transmission losses, but it will increase the waiting time after sources or loads are ready. The second aspect of the MPPT for the MISIMO is to compromise the waiting time and the ratio of one-stage transfer, and various algorithms have been proposed. In [52], the frequencies of the source-to-load and source-to-battery were the same, the energy transferred of these two paths was allocated according to the need of the loads. When the sources were weak, the loads were powered by a battery. Compared with purely two-stage transfer, the energy transmission efficiency was improved by 11–13%. In [53], the source-to-load had a higher priority than the source-to-battery. The control of the energy transfer of the sources was replaced by a trained oscillator instead of a comparator. Same as [52], the battery-to-load occurred when the sources cannot satisfy the loads. It achieved 18 nA quiescent current, 2460 dynamic range, and the efficiency of 87% when the input was 20 μW. An event-driven mode was adopted in [54] with the independent modulation control for each input source and output source. The source-to-load occurred if the source was ready to provide energy and meanwhile the load needed power supply, and the sources and loads were prioritized to obtain the optimal energy transfer path. The source-to-battery or battery-to-load occurred when only one of the sources and loads is ready. A 32 nA quiescent current, 1.2 × 10^5^ dynamic range, 3.2× energy extraction gain for the PEH, and 80% efficiency at 1 μA output current were achieved.

## 6. Conclusions

This brief discusses the state-of-the-art design techniques in achieving high efficiency piezoelectric energy harvesting systems. Through the introduction and modeling of the PEH, the important roles of the rectifiers and the non-linear methods to extract energy from the PEH to loads and improve the output power by realizing conjugate matching indirectly are discussed. The circuit techniques of the existing PEH interface circuits are introduced and summarized based on topologies of rectifier, non-linear methods and MPPT circuits. In terms of improving the AC-DC power conversion efficiency and meeting the system requirements, several methods to reduce the voltage drop, the reverse current and the startup time are introduced and compared. Furthermore, improving the flipping efficiency and the MPPT efficiency are also the effective approaches to improve the harvesting efficiency. Their characteristics such as the power level, the system volume and the power losses are introduced. The pros and cons of the corresponding representative techniques employed in PEH interfaces are summarized for comprehensive understanding.

## Figures and Tables

**Table 1:** Passive component parameters of the bimorph piezoelectric harvester T226-A4-503X.

*R_M_*	*L_M_*	*C_M_*	*C_P_*	*n*
15.51 Ω	1 H	12.13 μF	41.24 nF	−0.0196

**Table 2:** Comparison for different implementation of the rectifier.

	Voltage Drop	Power Efficiency	*V_RECT_*/*V_P_*	Self-Start
Diode-connected rectifier [10]	2 *V_TH_*	3 *V_DS_*	55% (1.1/2.2)	√
NVC [11]	3 *V_DS_*	86%	98.3% (1.77/1.8)	
Comparator-controlled [12]	2 *V_DS_*	87%, *R_L_* = 100 Ω	95% (1.9/2), *R_L_* = 2 kΩ	
Operational amplifier controlled [13]	2 *V_DS_*	90%, *R_L_* = 95 kΩ	99% (2.78/2.8)	
Fully comparator controlled [21]	2 *V_DS_*	95%, *R_L_* = 20 kΩ	99% (4.88/4.9), *R_L_* = 200 kΩ	
Dual-mode rectifier [24]	3 *V_DS_*/3 *V_DS_*	90%	98.8% (1.66/1.68)	√

**Table 3:** Comparison for the non-linear methods based on the SECE and the SSH.

	Method	P_OUT_	Components	MOPIR	Pros	Cons
[14]	SSHI	408 μW	*L* = 3.3 mH	4.93×	High output power, high MOPIR	Large high Q inductor is required
[1]	FCR	50.2 μW	* C_total_ = 1.44 nF (4 cap)	4.83×	High MOPIR, fully integrated	High control complexity
[38]	SSHCI	19 μW	*L* = 68 μH, * C_total_ = 453 nF (1 cap)	3.52×	Potential for higher output power than SSHI with the same inductor	Both extra inductor and capacitors needed
[27]	SECE	477 μW	*L* = 10 mH	1.23×	load independent output power	Relatively Low MOPIR
[17]	MCE	78 μW	*L* = 1 mH	2.1×	Lower conduction losses	High control complexity

* C_total_ = total capacitance of all capacitors except the output capacitor utilized for flipping.

**Table 4:** Comparison of the MPPT circuits based on the P&O and the FOCV.

	Method	Features	Efficiency	Pros	Cons
[26]	P&O	Independent from the PEH characteristics	94%	High power level (8.4 mW), MCU based computer	High power consumption and long detection time (110 μA, 120 s)
[42]	P&O		99.9%	High efficiency, fully analog computer (900 nA < I < 1.3 µA)	Relatively long detection time (<1 s)
[43]	P&O		97%	P&O for SSHI with 4.17x MOPIR (I = 430 nA)	Fixed step size
[37]	FOCV	Short time and low power consumption (0 to several cycles, dozens of nA)	95.7%	Detecting high VOC below withstand voltage	Stop harvesting during detection
[24]	FOCV		99%	Short detection time (one-cycle), 9.09 ms/V tracking	Stop harvesting during detection, additional peak detector required
[44]	FOCV		72–99%	Adaptive adjustment without disconnection	Relatively low accuracy

## Data Availability

Not applicable.
